# Safe suture anchor insertion for anterior and posterior hip labral repair

**DOI:** 10.1093/jhps/hnv027

**Published:** 2015-04-29

**Authors:** Andrew D. Foster, John Ryan, Thomas Ellis, James Flom

**Affiliations:** 1. Department of Orthopaedics, The Ohio State University Wexner Medical Center, Columbus, OH, USA; 2. Ortho One, Columbus, OH, USA; 3. Pivot Medical, Columbus, OH, USA

## Abstract

We sought to define bone thickness in relation to the chondral surface at various depths along the anterior and posterior acetabular rim and safe portals for anchor insertion in these regions. Six cadaveric pelvises were mounted on a custom jig. A custom guide was attached to simulate anterolateral (AL), mid-anterior (MA), distal anterolateral (DALA) and posterolateral (PL) arthroscopy portals. Anterior 3 o’clock and 4 o’clock positions were drilled using MA and DALA portals. Posterior 8 o’clock to 11 o’clock positions were drilled using a 1.4-mm drill bit from the PL portal. At depths of 5, 10 and 15 mm, the distance from the drill to the intra and extra-articular surfaces was measured using a custom caliper. Mean distance between drill hole and articular surface for anterior and posterior positions ranged from 1.61 to 2.75 mm at 5 mm. The smallest distance between the drill hole and articular surface and the largest width between drill hole and the extra-articular surface were at the 4 o’clock position. No difference between the MA and DALA portals were noted for the anterior positions. For the posterior rim positions, the distance on the articular side remained consistent throughout. For the posterior positions, only the PL portal was utilized. Both the MA or DALA portals can be utilized for safe drilling of the anterior rim positions. The posterior positions can all be safely drilled with a relatively good bone margin using the PL portal, but use of the MA or DALA portals resulted in extra-articular cortical perforation in all cases.

## INTRODUCTION

The labrum is an important structure for maintaining hip biomechanics. It increases the acetabular volume and creates a negative pressure seal within the hip joint when peripheral contact is established first [1–4]. The resistance of synovial flow results in a more uniform compressive load applied to the articular cartilage, efficiently provides nutrition to the chondrocytes, and provides fluid for a smooth gliding joint [5]. Thus, in addition to pain and instability, labral tears could lead to degenerative changes in the hip. Proposed mechanisms of this include microinstability, decreased cartilage nutrition, increased cartilage consolidation and reduced contact area.

 Labral tears are the most common pathology identified in patients undergoing hip arthroscopy and are the most common cause of mechanical symptoms [6]. In the past decade, improved arthroscopic techniques have allowed for suture anchor repair of the hip labrum. However, the procedure is technically challenging due to the restricted angles of anchor insertion, concavity of the acetabular articular surface and relatively narrow column of bone in the anterior and posterior acetabulum. Failure of anchor insertion can occur by penetration of the intra or extra-articular acetabulum. The goal of this study was to define the bone thickness in relation to the articular surface at various depths along the anterior and posterior acetabular rim and to define safe portals for anchor insertion in these regions.

## METHODS

**Fig. 1.**
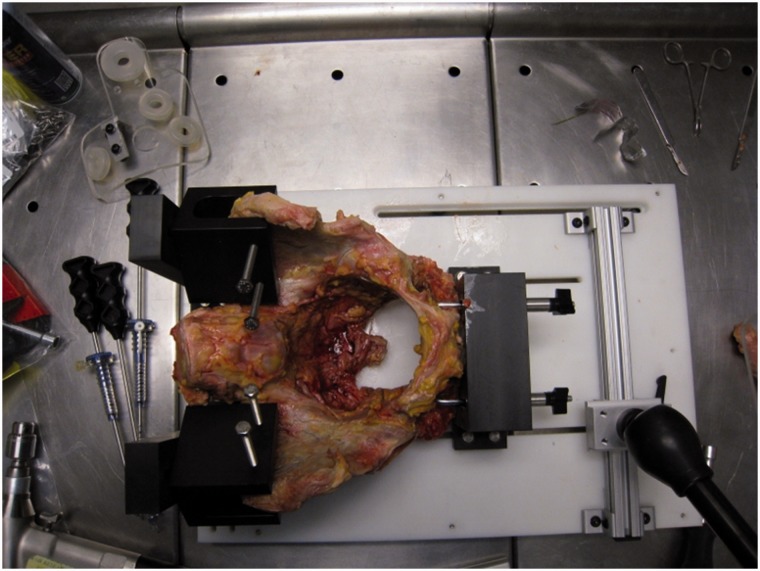
Cadaveric pelvis stripped of soft tissue, mounted onto jig.

**Fig. 2.**
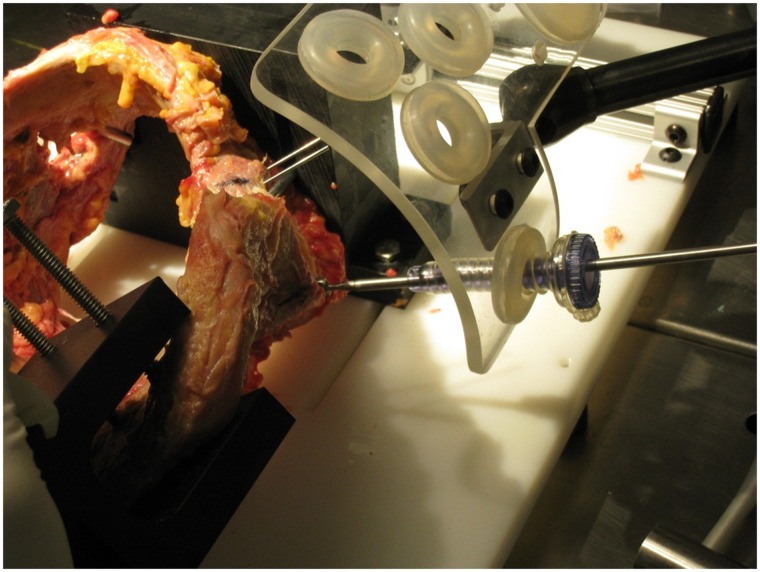
Custom guide simulating antero-lateral (AL), mid-anterior (MA), distal antero-lateral (DALA) and postero-lateral (PL) arthroscopy portals.

**Fig. 3.**
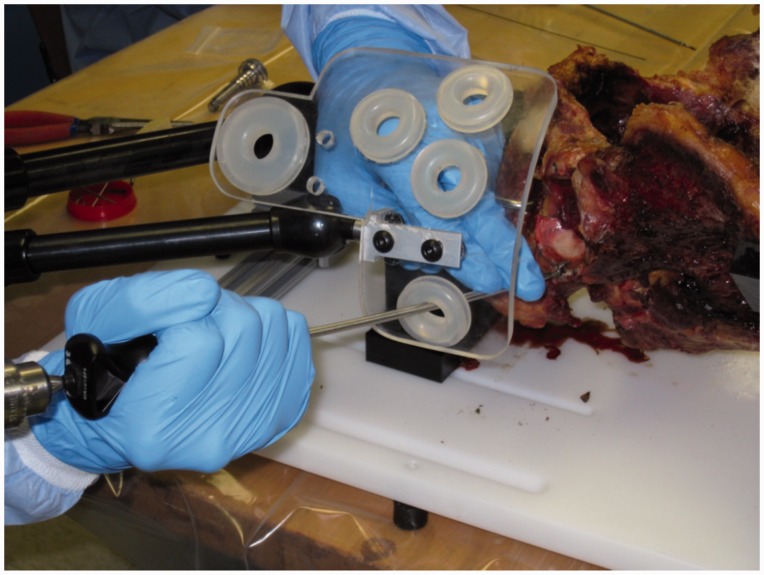
The DALA portal position was positioned relative to other portals based on the figure descriptions in Robertson *et al*. [8].

Six fresh frozen adult cadaveric pelvises (2 females, 4 males, age 29–56, average 44.5) were dissected free of the soft tissues, including the labrum. The right and left acetabuli were utilized. Each pelvis was mounted onto a jig (Fig. 1) with custom guide (Fig. 2) which simulated skin portals at each of four standard arthroscopy portals: anterolateral (AL), mid-anterior (MA), distal anterolateral (DALA) and posterolateral (PL). Portals were placed in the jig following the technique described by Byrd [7]. The portal positions were determined based on the article by Robertson *et al.* [8] (Fig. 3). Clock face positions were marked on the acetabuli with the midpoint of the transverse acetabular ligament denoting the 6 o’clock position.

**Fig. 4.**
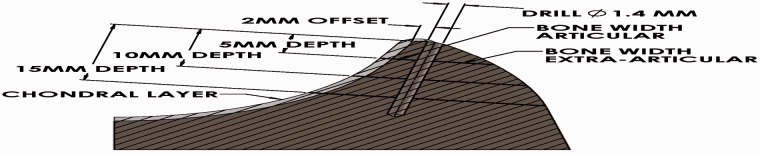
A 1.4-mm drill bit with 2 mm offset was used to simulate anchor insertion. Measurements of the articular bone width and extra-articular bone width were taken at 5, 10 and 15 mm from the labral insertion.

A 1.4-mm drill bit with a 2-mm offset straight drilling guide referenced from the articular surface was used to simulate typical anchor insertion. The guide is placed against the articular surface which places the drill bit such that the edge of the hole created is 2 mm from the reference surface. This was designed to place the anchor close to the articular surface. The MA and DALA portals were used to drill the 3 o’clock and 4 o’clock positions, and the PL portal was used to drill the 8 o’clock to 11 o’clock positions. A custom caliper was then used to measure the distance from articular chondral surface to drill (articular width) as well as the distance from drill to extra-articular surface (bone width, extra-articular, Fig. 4). The caliper included a 1.4-mm pin which was placed in the drilled hole, which also included etched marks to measure depth. Measurements were taken at 5, 10 and 15 mm depths from the labral insertion and measured parallel to the acetabular face, which was defined as the distance along a perpendicular line between drill and articular/extra-articular surface. Standard deviations were calculated.

## RESULTS

Mean distance from drill to articular chondral surface ranged from 1.61 to 2.75 mm in the various anterior and posterior positions at a depth of 5 mm (Table I). The actual distance is less than these measurements, as the measurements include articular cartilage thickness (Fig. 4). In most cases, the distance to the articular surface increases as drill depth increases.

**Table I.** Summary of measurements taken at various depths and rim positions

Twelve hip specimens		Articular side of acetabulum			Extra-articular side of acetabulum		
Portal	Clock position	Depth			Depth		
		5 mm	10 mm	15 mm	5 mm	10 mm	15 mm
MA	3	2.23 ± .65	3.31 ± 1.20	5.15 ± 1.90	3.18 ± 1.08	5.26 ± 1.65	6.05 ± 2.18
	4	1.62 ± .74	2.30 ± .96	1.39 ± 2.00	10.66 ± 2.27	12.95 ± 1.63	13.64 ± 2.44
DALA	3	2.60 ± .87	3.80 ± 2.37	6.59 ± 4.09	3.10 ± 2.14	4.84 ± 2.64	4.78 ± 2.82
	4	1.61 ± .48	1.98 ± 1.07	2.51 ± 2.42	7.94 ± 3.91	10.88 ± 3.78	11.47 ± 3.44
PL	8	2.73 ± .78	5.43 ± 1.76	8.55 ± 2.63	4.38 ± 1.57	7.23 ± 2.29	9.93 ± 3.77
	9	2.29 ± .64	4.70 ± 1.49	8.19 ± 2.13	4.04 ± 1.40	5.58 ± 1.95	6.15 ± 2.62
	10	2.10 ± .79	4.14 ± 1.40	7.45 ± 2.18	3.40 ± 1.92	4.96 ± 2.30	6.38 ± 2.75
	11	2.75 ± .80	5.34 ± 1.18	9.05 ± 2.21	3.28 ± 1.34	4.38 ± 1.52	5.44 ± 1.93

Values reported as averages and standard deviations.

**Fig. 5.**
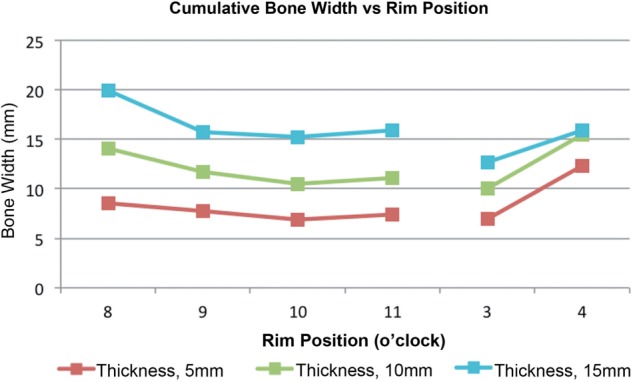
Cumulative bone width at various depths as related to acetabular rim position.

The total acetabular bone thickness was largest at the 4 o’clock and 8 o’clock positions (Fig. 5). Posterior positions demonstrate relatively constant articular distance from the drill bit (2.5/4.8/8.5 mm at 5/10/15 mm depths). For the posterior positions, the PL portal only was used. Use of the MA or DALA portals resulted in extra-articular surface perforation as drill penetration increased.

The smallest distance between the drill hole and articular surface and the largest distance between drill hole and the extra-articular surface were both at the 4 o’clock position. This was similar when measured from either the MA or DALA portal. No significant difference between the MA and DALA portals were noted for the anterior positions (Tables I and II). Unlike other rim positions, at which the distance to the articular surface increases with increasing depth, the distance to the articular surface at 4 o’clock remains relatively thin at 10 and 15 mm. In 6 of the 12 specimens, the drill hole penetrated into the acetabular fossa between 10 and 15 mm.

**Table II.** Comparisons performed between the MA and DALA portals

		Articular				Extra-articular			
Rim Loc.	Drill depth	DALA	MA	Mean difference	*P* value	DALA	MA	Mean Diff	*P* value
3	5	2.60	2.23	0.37	0.460	3.10	3.18	0.09	0.909
	10	3.80	3.31	0.49	0.664	4.84	5.26	0.42	0.728
	15	6.59	5.15	1.44	0.458	4.78	6.05	1.27	0.405
4	5	1.61	1.63	0.02	0.954	7.94	10.66	2.72	0.178
	10	1.98	2.30	0.32	0.602	10.88	12.95	2.07	0.264
	15	2.51	1.39	1.12	0.404	11.47	13.64	2.16	0.240

## DISCUSSION

Hernandez and McGrath were the first to study acetabular anatomy with respect to suture anchor placement for labral repair [9]. They measured the width of acetabular bone at labral insertion as well as 5, 10, 15 and 20 mm from the labral insertion at the 12 o’clock, 1:30 and 3 o’clock positions. They found no significant difference at more superficial levels between the three measured locations, however found the 3 o’clock position to be significantly wider at deeper (15 and 20 mm) depths. They suggested this corresponds to the relationship of the anterior acetabulum and the superior pubic ramus. They also noted especially small widths at the level of the labral insertion (less than 3 mm), and suggested using drills <3.0 mm for anchor insertion if the labral attachment is used as the starting point.

The anterosuperior labrum is the most common site of pathology, however locations of tears can vary. Lertwanich *et al*. [10] used 3D CT reconstruction models to measure acetabular rim angles from the 8 o’clock to 4 o’clock positions. This angle quantifies the angle between the subchondral margin and outer cortex of acetabular bone and is a surrogate measure of rim thickness. Measurements were taken at 10, 12.5, 15, 20 and 25 mm from three different anchor insertion sites to simulate different anchor or drill bit lengths and the effect of acetabuloplasty. The areas of highest acetabular rim angles were the 2 o’clock and 8 o’clock positions, corresponding to the anterior inferior iliac spine and the ischial tuberosity, respectively. The area of lowest acetabular rim angle was the 3 o’clock position, which represents the psoas valley. Their results were similar to Kohnlein *et al*. who evaluated spatial acetabular rim profile using plaster molds of 66 acetabuli and found prominences at the 1:50, 4:40 and 7:50 clock face positions [11]. However, their results were different than Hernandez and McGrath in that the 1 and 2 o’clock (anterosuperior) acetabular rim angles were significantly higher than the superior (12 o’clock) and anterior (3 o’clock) positions [9].

Our study confirmed the results of the Lertwanich *et al*. study, namely that anterior and posterior rim position have relatively thin bone [10]. Both the MA and DALA portals can be safely used for drilling anterior rim positions. At the 4 o’clock position, the distance from drill hole to articular surface remains quite thin throughout the entire drill depth. Surgeons should note the lower safety margin in this area when performing labral repair or reconstruction and consider using smaller diameter anchors in this region. Posterior positions can be safely drilled using the PL portal with relatively good bone margin. The MA and DALA portals should not be used when drilling posterior positions as this resulted in extra-articular surface penetration in our model. In all specimens, increased extraarticular bone is noted at the lower positions, corresponding to the ischial and pubic flare.

There are several limitations to our study. We did not analyze anchor placement from 12 to 2 o’clock. The acetabular bone is thick in this region as demonstrated by previous studies and therefore safe anchor placement is not problematic [7, 9, 10]. Also, the measurement technique for the distance from the drill to the articular surface included articular cartilage thickness, so our measurements actually overestimated the amount of subchondral bone. A 2-mm offset guide was used to standardize drilling the holes. In some positions, such as at 4 o’clock position, a larger offset might have resulted in increased articular distance, preventing joint penetration. However, this would risk pulling the labrum away from the rim as the suture is tightened. Finally, straight drill guides were used in this study making extrapolation of the results for curved/angled guides difficult.

## CONCLUSION

Our study emphasizes the need for precise anchor placement on the acetabular rim. Specifically, surgeons should be aware of the thin acetabular rim at the anterior and posterior positions, especially at 4 o’clock, where the distance to the articular surface remains small even with increasing depth. Surgeons can safely use either the MA or DALA arthroscopy portals for anterior labral repair and PL portal for posterior repairs.

## FUNDING

Pivot Medical, Sunnyvale, CA.

## CONFLICT OF INTEREST STATEMENT

None declared.
